# Rare confounder: benign multicystic peritoneal mesothelioma in a patient with mucinous colon adenocarcinoma

**DOI:** 10.1093/jscr/rjae569

**Published:** 2024-09-05

**Authors:** Kruti K Patel, Griffin P Stinson, Krista P Terracina, Johan F Nordenstam, Thomas E Read

**Affiliations:** Division of Gastrointestinal Surgery, University of Florida College of Medicine, 1600 SW Archer Road, Rm 6165, PO Box 100109, Gainesville, FL 32601, United States; Division of Gastrointestinal Surgery, University of Florida College of Medicine, 1600 SW Archer Road, Rm 6165, PO Box 100109, Gainesville, FL 32601, United States; Division of Gastrointestinal Surgery, University of Florida College of Medicine, 1600 SW Archer Road, Rm 6165, PO Box 100109, Gainesville, FL 32601, United States; Division of Gastrointestinal Surgery, University of Florida College of Medicine, 1600 SW Archer Road, Rm 6165, PO Box 100109, Gainesville, FL 32601, United States; Division of Gastrointestinal Surgery, University of Florida College of Medicine, 1600 SW Archer Road, Rm 6165, PO Box 100109, Gainesville, FL 32601, United States

**Keywords:** benign multicystic peritoneal mesothelioma, colon adenocarcinoma

## Abstract

Benign multicystic peritoneal mesothelioma (BMPM) is a rare condition, in which patients have multiple cystic lesions of the peritoneum. BMPM can mimic mucinous carcinomatosis and can thus create a diagnostic dilemma. We present the case of a 76-year-old woman who was referred for management of ascending colon adenocarcinoma and was noted to have several nonspecific cystic lesions in the abdomen and pelvis on preoperative computed tomography and diagnostic laparoscopy. Frozen section analysis suggested the lesions contained ‘mucin’. Due to concern for metastases, right colectomy was aborted. Final histologic analysis of the laparoscopic biopsies revealed mesothelial cysts, consistent with BMPM, unrelated to her colon adenocarcinoma. Laparoscopic right colectomy was performed 2 weeks later. BMPM can create diagnostic and therapeutic uncertainty in patients with known visceral malignancies when discovered incidentally. Frozen section analysis may not be accurate in differentiating the two, and final histologic confirmation should be sought prior to definitive treatment.

## Introduction

Benign multicystic peritoneal mesothelioma (BMPM) is a rare condition, with fewer than 200 known cases reported in the medical literature [1]. It is characterized by multiple cystic lesions of the peritoneum that carry a low risk of malignancy. It most commonly presents in pre-menopausal women with very few cases reported in post-menopausal women, or in men [2]. Risk factors are thought to include endometriosis, pelvic inflammatory disease, and prior abdominal surgery. The pathogenesis of this condition remains unclear, with some theories suggesting multicystic peritoneal mesothelioma develops in response to chronic irritation or inflammation of the peritoneum [1]. It should be noted that benign multicystic mesothelioma is unrelated to asbestos exposure, in contrast to pulmonary mesothelioma [3].

Clinically, patients may present with abdominal discomfort or distention, but most cases are discovered incidentally during abdominal imaging or abdominal exploration for unrelated conditions. BMPM can often mimic other peritoneal conditions, and imaging studies are often unable to differentiate it from other possible lesions. Herein, we report a case where BMPM mimicked mucinous carcinomatosis, creating a diagnostic dilemma in a patient undergoing treatment of mucinous adenocarcinoma of the ascending colon.

## Case report

A 76-year-old woman was referred for management of ascending colon adenocarcinoma. Her past surgical history was remarkable for bilateral salpingo-oophorectomy 20 years prior, cesarean section, and a left sided Spigelian hernia repair with intraperitoneal mesh. There was no personal or family history of colon cancer, Lynch syndrome, or polyposis. She reported a history of abdominal ‘cysts’ that were discovered incidentally at hysterectomy although no operative or pathology reports from that operation were available.

**Figure 1:**
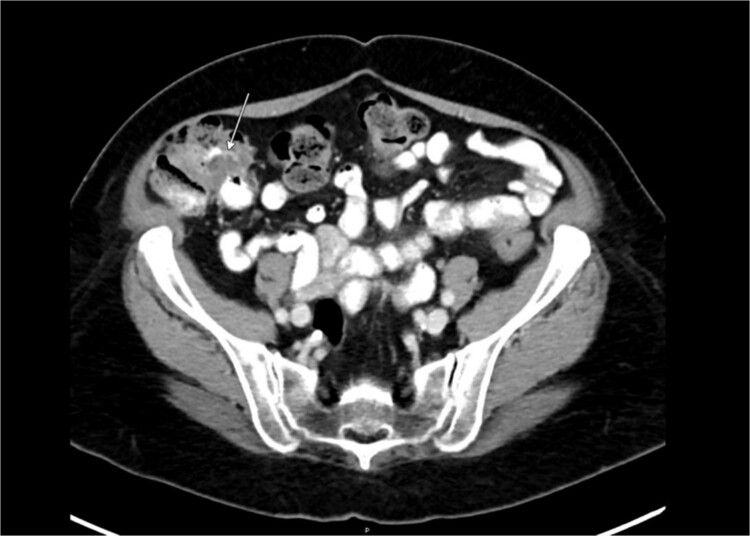
Computed tomography abdomen/pelvis—arrow indicates ascending colon mass.

**Figure 2:**
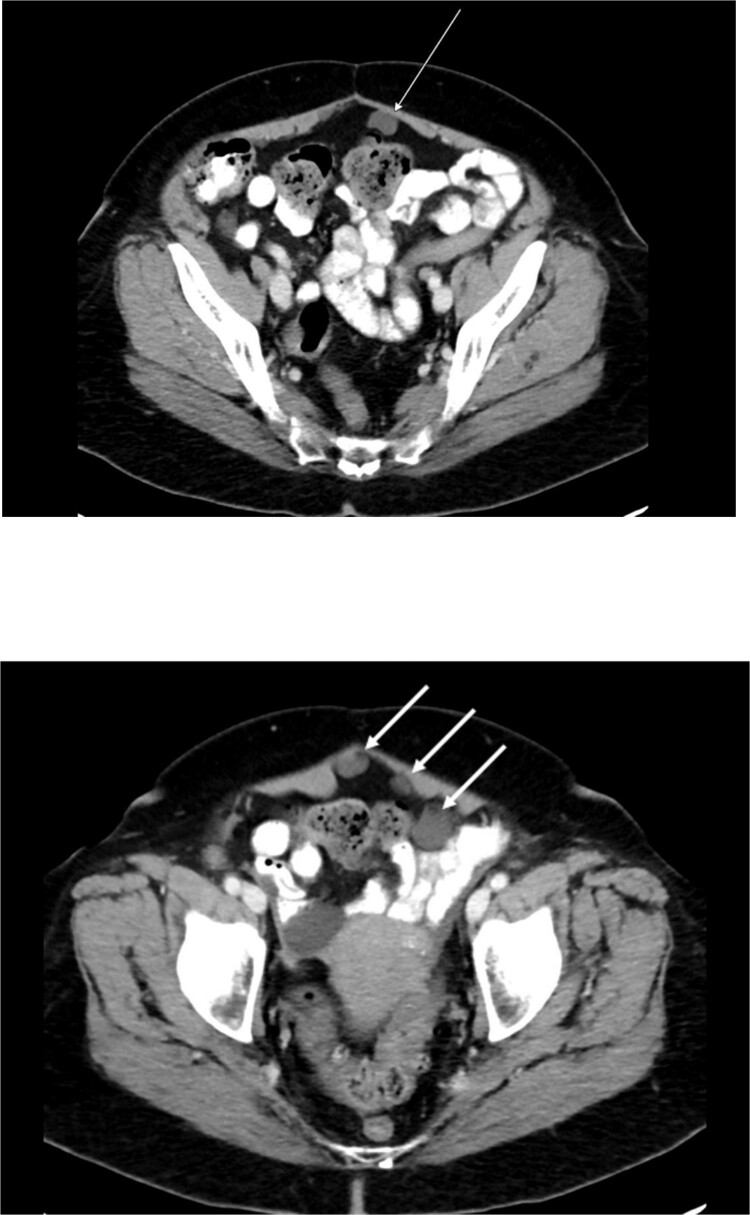
Computed tomography abdomen/pelvis—arrows indicate peritoneal cysts.

Preoperative abdominal computed tomography revealed a mass in the ascending colon (Fig. 1) and multiple nonspecific cystic lesions in the peritoneal cavity involving the abdomen and pelvis (Fig. 2). Normally, carcinomatosis would be suspected in a patient with mucinous adenocarcinoma of the colon. However, the radiographic findings were not definitive for carcinomatosis, and the history of abdominal ‘cysts’ 20 years previously further confused the situation. The differential at the time included malignant carcinomatosis, benign endometriotic cysts, and cystic peritoneal reaction to previously placed intraperitoneal hernia mesh. Carcinoembryonic antigen measured 0.8 ng/mL.

**Figure 3:**
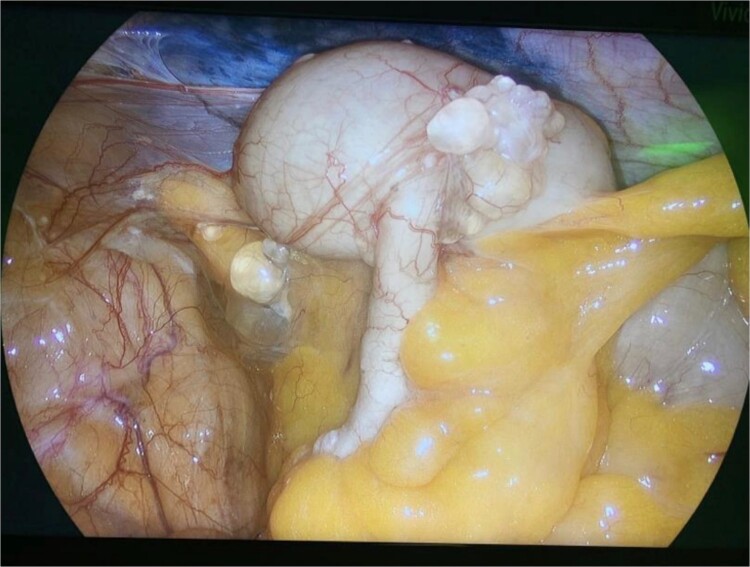
Intraoperative view of the right lower quadrant of the abdomen: cysts involving peritoneum of the right colon and mesentery.

**Figure 4:**
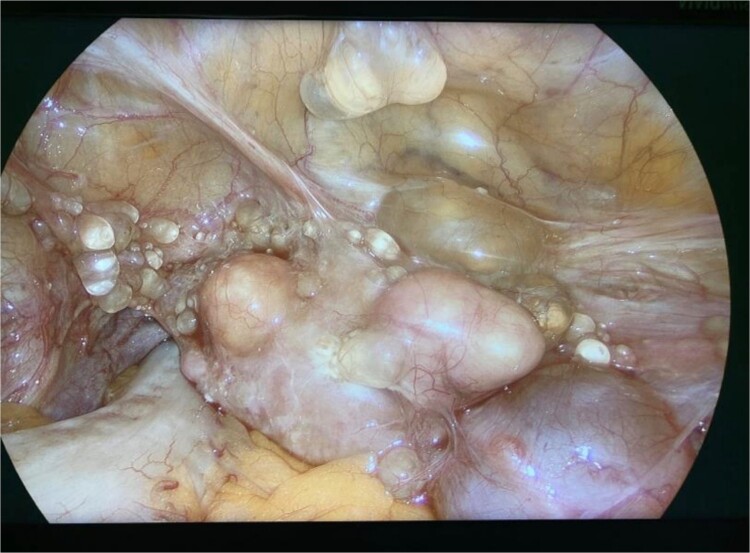
Intraoperative view of the pelvis: multiple cysts involving the peritoneum.

Diagnostic laparoscopy demonstrated multiple cystic lesions throughout the abdomen and pelvis (Figs 3 and 4). The gross appearance of these cysts was consistent with mucinous carcinomatosis. Laparoscopic excisional biopsy of several lesions was performed. Frozen section analysis revealed mucin but did not reveal evidence of metastatic adenocarcinoma. Colectomy was deferred due to concern for carcinomatosis, as if confirmed, she would likely be treated with neoadjuvant chemotherapy, restaging, followed by cytoreductive surgery (to include synchronous right colectomy) with heated intraperitoneal chemotherapy.

However, the frozen section results were not confirmed on final histologic analysis of the excised lesions. Rather, the cystic lesions were found to contain mesothelial cysts, consistent with BMPM, unrelated to the patient’s primary colon adenocarcinoma.

The patient underwent laparoscopic-assisted right colectomy with ileocolic anastomosis 2 weeks after initial diagnostic laparoscopy. Her postoperative course was uncomplicated, and she was discharged home on postoperative Day 3. Histologic evaluation of her right colon specimen revealed a pT2N0 (Stage I) mucinous adenocarcinoma with negative margins and no adverse histologic features.

The patient provided informed consent for the collection and publication of information related to this case.

## Discussion

As demonstrated in this case, BMPM may create a diagnostic dilemma, as its gross and radiographic appearance often mimics carcinomatosis. This is especially true in patients being treated for visceral adenocarcinoma. Patients may undergo needless treatments, or have appropriate treatment withheld, because of the mistaken radiographic diagnosis of carcinomatosis [4].

The diagnosis is often not considered when patients are being staged prior to initiation of treatment for visceral malignancy, and it is challenging to differentiate BMPM from carcinomatosis radiographically. Thus, surgical excisional biopsy and histologic analysis may be required for definitive diagnosis. Frozen section analysis may not be adequate to make the diagnosis, as illustrated in this case, where BMPM was mistaken for mucinous implants on frozen section.

Treatment options for benign multicystic mesothelioma include observation and excision, although recurrence rates of up to 50% after excision have dampened enthusiasm for excision in asymptomatic patients [3, 5]. Given the benign appearance of the lesions histologically, and her remote history of ‘abdominal cysts’ that have not caused her symptoms, we elected to follow her expectantly for this condition.
